# Retracted: Differential proteomic profiling to study the mechanism of cardiac pharmacological preconditioning by resveratrol

**DOI:** 10.1111/j.1582-4934.2012.01620.x

**Published:** 2012-09-26

**Authors:** Karel Bezstarosti, Samarjit Das, Jos M J Lamers, Dipak K Das

**Affiliations:** a Cardiovascular Research Center, University of Connecticut School of MedicineFarmington, CT, USA; b Department of Biochemistry Cardiovascular Research School COEUR, Erasmus Medical CenterRotterdam, The Netherlands

The following article from *Journal of Cellular and Molecular Medicine* (volume 10, issue 4, 2006, pages 896–907), ‘Differential proteomic profiling to study the mechanism of cardiac pharmacological preconditioning by resveratrol’ by Karel Bezstarosti, Samarjit Das, Jos. M. J. Lamers and Dipak K. Das, and published in the October 2006 issue in Wiley Online Library (http://onlinelibrary.wiley.com), has been retracted by agreement between the authors, the journal Editor-in-Chief, Professor LM Popescu and Blackwell Publishing Ltd. The retraction has been agreed due to findings of data fabrication in the western blot data in Figure 6 that occurred in the laboratory of Dipak K. Das in an investigation into the work of Dipak K. Das by the University of Connecticut Health Center. Although no questions have been raised concerning the data generated by Karel Bezstarosti and Jos M J Lamers, these authors fully support the retraction.
